# Impact of Caloric Restriction and Exercise on Trimethylamine N-Oxide Metabolism in Women with Obesity

**DOI:** 10.3390/nu15061455

**Published:** 2023-03-17

**Authors:** Daniel J. Battillo, Steven K. Malin

**Affiliations:** 1 Department of Kinesiology and Health, Rutgers University, New Brunswick, NJ 08901, USA; 2 Department of Kinesiology, University of Virginia, Charlottesville, VA 22903, USA; 3 Division of Endocrinology, Metabolism & Nutrition, Rutgers University, New Brunswick, NJ 08901, USA; 4 New Jersey Institute for Food, Nutrition and Health, Rutgers University, New Brunswick, NJ 08901, USA; 5 Institute of Translational Medicine and Science, Rutgers University, New Brunswick, NJ 08901, USA

**Keywords:** trimethylamine N-oxide, low-calorie diet, interval exercise, obesity, hemodynamics, cardiovascular disease

## Abstract

Trimethylamine N-oxide (TMAO) is linked to cardiovascular disease (CVD) through partly altered central hemodynamics. We sought to examine if a low-calorie diet plus interval exercise (LCD+INT) intervention reduces TMAO more than a low-calorie diet (LCD) program alone in relation to hemodynamics, prior to clinically meaningful weight loss. Women with obesity were randomized to 2 weeks of LCD (*n* = 12, ~1200 kcal/d) or LCD+INT (*n* = 11; 60 min/d, 3 min at 90% and 50% HRpeak, respectively). A 180 min 75 g OGTT was performed to assess fasting TMAO and precursors (carnitine, choline, betaine, and trimethylamine (TMA)) as well as insulin sensitivity. Pulse wave analysis (applanation tonometry) including augmentation index (AIx75), pulse pressure amplification (PPA), forward (Pf) and backward pressure (Pb) waveforms, and reflection magnitude (RM) at 0, 60, 120, and 180 min was also analyzed. LCD and LCD+INT comparably reduced weight (*p* < 0.01), fasting glucose (*p* = 0.05), insulin tAUC_180min_ (*p* < 0.01), choline (*p* < 0.01), and Pf (*p* = 0.04). Only LCD+INT increased VO_2_peak (*p* = 0.03). Despite no overall treatment effect, a high baseline TMAO was associated with decreased TMAO (r = −0.45, *p* = 0.03). Reduced TMAO was related to increased fasting PPA (r = −0.48, *p* = 0.03). Lowered TMA and carnitine correlated with higher fasting RM (r = −0.64 and r = −0.59, both *p* < 0.01) and reduced 120 min Pf (both, r = 0.68, *p* < 0.01). Overall, treatments did not lower TMAO. Yet, people with high TMAO pre-treatment reduced TMAO after LCD, with and without INT, in relation to aortic waveforms.

## 1. Introduction

Cardiovascular disease (CVD) is a leading cause of death worldwide [1]. The gut microbiome has recently been implicated as an important mediator of atherosclerosis and vascular dysfunction. One CVD risk mediator that has garnered particular attention is trimethylamine N-oxide (TMAO). TMAO is a gut-derived metabolite produced largely from dietary sources such as meat, fish, and eggs that are rich in choline, carnitine, and betaine. These later metabolites are metabolized by gut bacteria to form trimethylamine (TMA) via TMA lyase [2]. In turn, TMA is oxidized in the liver by hepatic flavin monooxygenases, particularly FMO3, to form TMAO [3]. High TMAO levels are clinically concerning because it is linked to foam cell production, inflammation, and endothelial dysfunction, all of which promote arterial stiffness [3,4,5]. Indeed, TMAO has been associated with obesity, type 2 diabetes, and cardiovascular disease as well as hypertension [6,7,8]. However, identification of optimal therapeutic means to counteract TMAO levels and CVD risk remains unclear.

Lifestyle modification consisting of diet and exercise are first line therapies for reducing CVD risk. Dietary intake has been a primary interest in reducing TMAO production and concentration, given the association between carnitine and choline consumption with TMA metabolism by bacteria in the gut. In particular, diets low in red meat reduce plasma TMAO, and individuals adhering to vegetarian diets demonstrate lower TMA [9,10]. Not surprisingly, hypocaloric diets have also been proposed to reduce TMAO, through both lower animal product consumption and overall caloric restriction [11]. Alternatively, aerobic exercise may reduce CVD risk through improvements to gut health and microbiota [12,13]. Some exercise studies, including [14], but not all [15] report reduced plasma TMAO levels. The discrepancy between exercise studies on TMAO is unclear but increased moderate to vigorous physical activity is associated with lower TMAO levels in older individuals [16]. These later findings suggest exercise may act to lower TMAO through a distinct fitness-related mechanism, compared with diet. Interestingly, Erikson et al. [14] showed that a hypocaloric diet plus exercise for 12 weeks lowered TMAO more effectively than exercise alone in older men and women. However, weight loss was significantly greater following diet plus exercise, thereby confounding whether the diet per se or additional weight loss promoted TMAO reduction. Further, both men and women were studied, and recent work highlights the potential of sex differences in the gut microbiome [17]. We recently reported that a low-calorie diet (LCD) was similarly effective at reducing aortic waveforms when compared with an LCD plus interval exercise (LCD+INT) treatment matched on energy availability, in middle-aged women [18]. Whether TMAO metabolism improves comparably following LCD or LCD+INT, in relation to vascular function in middle-aged women, is unknown. Therefore, we tested the hypothesis that LCD+INT would reduce plasma TMAO comparably to LCD, and this change in TMAO metabolism would relate to improved central hemodynamics.

## 2. Methods

*Participants.* Twenty-three sedentary women with obesity (48.4 ± 2.4 yr; 37.9 ± 1.4 kg/m^2^; Table 1) were recruited from the local community via advertisements. Individuals were excluded if physically active (>60 min/wk), pregnant or nursing, on medications known to affect glucose metabolism (e.g., biguanides, insulin, TZDs, etc.) and/or blood pressure (e.g., ACE-inhibitors, beta-blockers, etc.), participated in smoking with the previous 2 years, or had an unstable weight over the prior six months (>2 kg variation). Menses status was documented (LCD: 5 post-menopausal, 2 irregular menses; LCD+INT: 7 post-menopausal, 1 irregular menses) but not controlled for, provided no women took hormone replacement therapy. Further, all participants underwent fasted blood work and a routine physical to confirm no indication of chronic disease (e.g., renal, hepatic, and cardiovascular) and participation safety. Individuals provided written and verbal informed consent before participation as approved by the University of Virginia Institutional Review Board (IRB # 18316).

*Body Composition and Aerobic Fitness.* Fat mass and fat-free mass (FFM) were determined using air displacement plethysmography (BodPod, Concord, CA, USA), and waist circumference (WC) was measured 2 cm above the umbilicus using a soft tape measure. Peak oxygen consumption (VO_2_peak) and heart rate (HRpeak) were determined using a continuous incremental cycle ergometer test and indirect calorimetry (Carefusion, Vmax CART, Yorba Linda, CA, USA). VO_2_peak criteria included a cadence < 60 rpm, RER > 1.1 and volitional fatigue.

*Oral Glucose Tolerance Test*. Participants reported to the Clinical Research Unit (CRU) after an approximate 10 hr overnight fast. Individuals were instructed to refrain from strenuous exercise, medications, caffeine, and alcohol consumption for 24 h. An intravenous catheter was placed in the right antecubital fossa for blood draws to determine glucose and hormonal responses during a 75 g oral glucose load. Fasting blood was collected to measure levels of TMAO, TMA, carnitine, betaine, and choline. Blood draws were subsequently collected at 30, 60, 90, 120, and 180 min to measure glucose tolerance and insulin sensitivity as estimated by total area under the curve (tAUC) calculations using the trapezoidal model. Aortic waveforms were measured at 0, 60, 120, and 180 min (*see details below*). Post-intervention assessments were obtained about 24 h after the last training session.

*Pulse Waveform Analysis*. The SphygmoCor XCEL system (AtCor Medical, Itasca, IL, USA) was used to characterize hemodynamic and aortic waveform responses as previously described [18]. This characterization included brachial systolic (bSBP), diastolic (bDBP), and pulse pressure (bPP), central systolic (cSBP), diastolic (cDBP), and pulse pressure (cPP), heart rate (HR), augmentation pressure (AP), and index (AIx) as well as wave convolution aspects of forward (Pf) and backward (Pb) pressure and reflection magnitude (RM). Pulse pressure amplification (PPA) was calculated as the ratio of bPP to cPP. Augmentation index was corrected to a standard HR of 75 bpm using the manufacturer’s software. All measurements were obtained while individuals were laying quietly in the semi-supine position in a temperature-controlled room.

*Low-Calorie Diet*. Participants were instructed to record their ad-libitum dietary intake for 3 days prior to pre-intervention testing. Subjects underwent 13-day LCD (1000–1200 kcal/d) based on pre-operative diets recommended to obese adults undergoing bariatric surgery. Meal replacement shakes were given to participants at breakfast and lunch (Ensure^®^ Abbott Laboratories, USA, 8 fl. Oz; providing 160 kcal, 16 g protein, 2 g fat, 19 g CHO). Menus detailing options for low-kcal snacks and dinners not exceeding 600 kcal (e.g., lean protein with vegetables) were provided. To assess compliance and caloric intake, 13-day food records were assessed and averaged from the course of the intervention. Empty shake containers were also collected to verify consumption. Additionally, food logs were recorded in the 3 days preceding clinical testing before and after the intervention. Food intake was assessed using ESHA (Version 11.1, Salem, OR, USA), and pre- and post-intervention changes are reported.

*Exercise Training.* Participants randomized to LCD+INT completed 12 supervised INT sessions over 13 days. Exercise duration was progressively ramped up, such that participants completed 30 and 45 min of INT on day 1 and 2, respectively, and 60 min of exercise per session thereafter, with one rest day over the 13 days. Exercise sessions consisted of subjects cycling for 3 min at 50% heart rate peak (HRpeak) to warm up, followed by alternating 3 min periods of cycling at 90% and 50% of HRpeak for the 60 min session as previously described [18]. Participants completed a light 5 min cooldown on the cycle to facilitate HR recovery. The study team completed daily check-ins with participants to ensure they were not experiencing excessive soreness or overuse injuries. A mixed-meal shake (Ensure^®^ Abbott Laboratories, USA, 8 fl. Oz; providing 350 kcal, 13 g protein, 11 g fat, 50 g CHO) was provided after each exercise in efforts to equate energy availability between treatments.

*Biochemical Analysis.* Plasma glucose was measured immediately following collection using the glucose oxidase method (YSI Instruments 2300, Yellow Spring, OH, USA). Fasting blood samples were collected in EDTA tubes and centrifuged at 4 °C for 10 min at 3000 RPM. All bloods were frozen at −80 °C until further analysis. Plasma betaine, choline, carnitine, TMA, and TMAO were determined by liquid chromatography tandem mass spectrometry as described by Koeth et al. [19] and Kirsh et al. [20]. Data acquisition of TMAO metabolism was carried out using selective ion monitoring, and the concentration of each analyte was calculated against an 8-point standard curve for that analyte.

*Statistical Analysis.* Data were analyzed using GraphPad Prism version 9 (GraphPad Software, San Diego, CA, USA). Normality was assessed using the Shapiro–Wilk test, and non-normally distributed data were log-transformed for analysis. Baseline differences between groups were analyzed using Student’s unpaired t test. Repeated measures analysis of variance (ANOVA) was used to determine group x time differences. Change in TMAO pre-intervention to post-intervention was calculated, and participants were categorized as either responders (decreased plasma TMAO) or non-responders (increased plasma TMAO) following their respective interventions. There were no baseline or post-intervention differences in variables of interest between the responder and non-responder groups. Pearson or Spearman rank correlations were used to assess normally and non-normally distributed outcomes, respectively. Statistical significance was accepted as *p* ≤ 0.05 and data are presented as mean ± SEM.

## 3. Results

*Participant Characteristics and Diet.* LCD and LCD+INT comparably reduced weight and body fat (all *p* < 0.01; Table 1). There were no significant changes in FFM following either treatment (*p* = 0.78; Table 1). Additionally, only LCD+INT increased VO_2_peak, compared to a slight decrease in LCD (*p* = 0.03; Table 1). LCD and LCD+INT reduced both fasting glucose and fasting insulin comparably (*p* = 0.05 and *p* = 0.03, respectively; Table 1), and each treatment reduced insulin tAUC_180min_ (*p* < 0.01; Table 1). While both groups decreased fasting LDL cholesterol (*p* < 0.01), only LCD+INT increased HDL cholesterol, compared to a reduction in LCD (*p* < 0.01). Both treatments reduced caloric intake similarly (*p* < 0.01), that was explained by reductions in carbohydrates (*p* = 0.05), fat (*p* < 0.01), and protein (*p* = 0.03, Table 2).

*Hemodynamics*. Fasting Pf was lowered after both LCD and LCD+INT (*p* = 0.04; Table 3), independent of changes in RM (*p* = 0.45) and AIx75 (*p* = 0.28). There were no changes in fasting cSBP and cDBP (*p* = 0.48 and *p* = 0.30, respectively; Table 3) or bSBP and bDBP (*p* = 0.47 and *p* = 0.39, respectively; Table 3), although there was a trending reduction in AIx75 tAUC (*p* = 0.08)_180min_ following each intervention. Further, there was no difference in fasting PPA (*p* = 0.90), but a trending reduction in fasting HR (*p* = 0.08; Table 3), following each intervention.

*TMAO Metabolism.* There were no differences in TMAO (*p* = 0.74), TMA (*p* = 0.62), betaine (*p* = 0.54), or carnitine (*p* = 0.89) following either intervention, whereas choline was reduced after LCD and LCD+INT (*p* < 0.01; Table 4).

**Figure 1:**
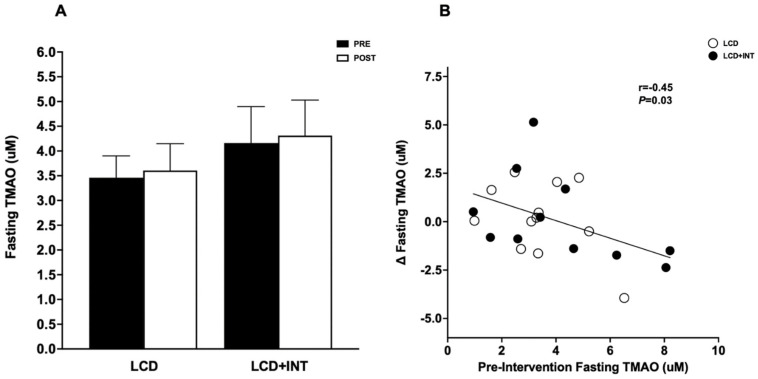
Effect of low-calorie diet (LCD) and low-calorie diet and exercise (LCD+INT) on fasting TMAO, and the correlation between pre-intervention fasting plasma TMAO and fasting TMAO changes following the intervention. Effect of low-calorie diet (LCD) and low-calorie diet and exercise (LCD+INT) on fasting TMAO levels. LCD and LCD+INT on TMAO levels (**A**). Correlation between pre-intervention plasma TMAO and TMAO changes following the intervention (**B**). Data are mean ± standard error of the mean (SEM).

**Figure 2:**
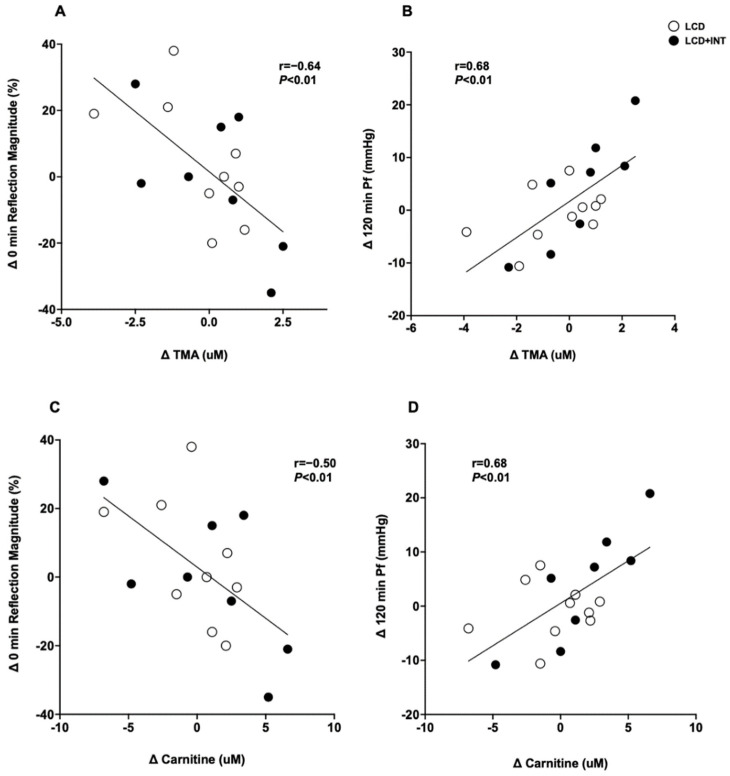
Correlations in TMA and carnitine changes following the intervention. Correlations in fasting TMA and carnitine changes following the intervention. The change (∆) in fasting TMA to the ∆ in 0 min reflection magnitude (RM) (**A**) and the ∆ in 120 min Pf (**B**). The ∆ in fasting carnitine to the ∆ in 0 min RM (**C**) and the ∆ in 120 min Pf (**D**).

Interestingly, a higher baseline TMAO was associated with greater reductions in TMAO following both LCD and LCD+INT (r = −0.45, *p* = 0.03; Figure 1). Furthermore, decreased TMAO was associated with increased fasting PPA (r = −0.48, *p* = 0.03). Reductions in fasting carnitine correlated with increased fasting RM (r = −0.59, *p* < 0.01) as well as lowered 120 min Pf (r = 0.68, *p* < 0.01). Similarly, lowered fasting TMA was also linked to reduced 120 min Pf (r = 0.68, *p* < 0.01) and greater fasting RM (r = −0.64, *p* < 0.01; Figure 2). Additionally, older age was associated with higher fasting TMAO before (r = 0.58, *p* < 0.01) but not after the intervention (r = −0.26, *p* = 0.23).

## 4. Discussion

The primary finding from this present study is that neither LCD nor LCD+INT was effective overall at reducing plasma TMAO in women with obesity, despite lowering plasma choline. However, we did observe that both treatments were effective at reducing TMAO in women with higher baseline levels. This is consistent with prior work demonstrating that participants with a median plasma TMAO level below 4.72 μM were free of CVD [21,22]. Thus, it is not entirely surprising that our lifestyle treatment had less robust changes across participants, given the average levels were 3.46 ± 0.4 μM and 4.16 ± 0.7 μM in LCD and LCD+INT, respectively. These discrepancies are interesting since other work has demonstrated that both caloric restriction and specific macronutrient-targeted diets are effective at reducing circulating TMAO [11,14,23]. However, this previous dietary work was mainly focused on vegan or low-fat diets, which potentially reduced animal product consumption and subsequent choline and carnitine intake more than the present study. While both groups in the present study saw reductions in all macronutrients, it is difficult to discern the extent of our observed dietary alterations, compared to these other studies [23,24]. Nonetheless, we did detect statistical reductions in circulating choline concentrations, which suggests that caloric restriction contributes to reduced TMAO precursor metabolites that influence TMAO. Indeed, our data are consistent with caloric restriction reducing choline levels in individuals with overweight and obesity [25]. This highlights that other precursors or factors may drive TMAO or have compensated to maintain TMAO levels. Regardless, in the present study, we had posited that exercise would augment the effect of an LCD to reduce TMAO, given some [16] studies demonstrated that exercise had a beneficial effect on plasma TMAO levels. Despite women cycling for 60 min a day over about 2 weeks in the present study, we did not detect statistical changes in TMAO. While longer-term exercise interventions may be required to elicit reductions in TMAO, it is worth noting that Erikson et al. [14] also did not detect changes in TMAO following 12 weeks of aerobic exercise training in older adults. Further, recent cross-sectional work in aerobically fit versus unfit individuals did not report TMAO differences [15]. It is difficult to reconcile why exercise studies are mixed on reducing TMAO, but our work points towards pre-treatment circulating TMAO concentrations as an important factor. Indeed, the reduction in TMAO was associated with increased fasting PPA. This association is clinically relevant as higher PPA suggests reduced central pulse pressure, compared with brachial pulse pressure, thereby reducing workload of the heart to propel blood into systemic circulation [26].

TMAO precursors are relevant to disease development, as choline, TMA, and carnitine levels have been associated with increased CVD risk [19,27,28]. Interestingly, LCD and LCD+INT similarly reduced plasma choline in the present study. The relevance of this reduction, though, is unknown, given lower choline levels did not associate with changes in aortic waveforms or blood pressure. It is worth noting that the relationship between dietary choline and plasma choline can differ among individuals, given the wide diversity of gut microbiota [29]. Therefore, it is possible that our LCD may have impacted how dietary choline was metabolized or that choline alone was not of sufficient concentration to influence vascular outcomes. In either case, reductions in TMA and carnitine were related to lower fasting RM after both treatments. RM is a ratio of the backward reflected wave (Pb) to the forward reflected wave (Pf). A higher RM suggests reduced effort of the heart to pump blood to the periphery [30]. In the present study, we observed a significant reduction in fasting Pf in both groups but no change in Pb. Collectively, with no change in Pb, these outcomes suggest that lower TMAO precursors may relate to a decreased left ventricular ejection fraction to support cardiac muscle function [31]. Conversely, it is important to recognize that the effect of carnitine is somewhat equivocal, compared to TMA, since it has been purported to reduce oxidative stress [32,33] and lower systolic and mean arterial pressures [34]. In fact, carnitine supplementation can increase left ventricular ejection fraction in patients with cardiomyopathy [35,36]. In our study, these later observations would be consistent with lower carnitine being related to lower RM and 120 min Pf as well as AIx75 tAUC_180min_. Since there were not equivalent reductions in brachial blood pressure after either treatment, our work highlights that the changes we see are likely at the level of the heart, rather than the peripheral vasculature. Somewhat surprisingly, post-prandial heart rate and bDBP tAUC_180min_ tended to rise after LCD+INT, compared to reductions following LCD. While exercise may have unique influences on central hemodynamics, compared with LCD, to maintain blood pressure during the fed state [37], these collective data highlight that reductions in TMA, choline, and carnitine appear important for aortic waveforms after lifestyle treatment in women. This observation is clinically relevant to targeting TMAO and its precursors in CVD etiology, as it conveys that TMAO has both central and peripheral effects on heart function and hemodynamics in women.

This study has limitations that may influence our interpretations. The present investigation only included women, so the results may not be generalizable to men. In fact, studies with similarly aged male and female cohorts have demonstrated lower TMAO levels in females than men [38] and may help explain the lower TMAO levels on average. Additionally, our sample size is modest, despite other lifestyle investigations on plasma TMAO that used similar sized cohorts and reported that lifestyle reduced TMAO [11,14]. Aging is also a consideration of plasma TMAO levels, as TMAO has been demonstrated to increase with age, independent of other CVD risk factors (e.g., systolic blood pressure and carotid–femoral PWV) [2,39]. As such, menopausal status should be considered in future work. In line with TMAO correlating with age at baseline (r = 0.58, *p* < 0.01), post-menopausal women would be anticipated to have more TMAO than premenopausal women. While this difference in menopausal status could influence ability to identify treatment effects, there was no association with age and the change in TMAO after the intervention. This suggests both LCD and LCD+INT potentially mitigate age-related CVD risk, regardless of menopausal status. Further, we were not able to quantify dietary intake of precursors such as choline, carnitine, and betaine in the study dietary logs due to technical difficulties with the software. Nevertheless, a strength of the study is that we were able to characterize TMAO-related precursors that have not been previously reported. Another consideration is that we only measured TMAO and its precursors in the fasted state of a 75 g OGTT. However, 5 days of a high-fat diet did not influence fasting or post-prandial TMAO levels in either sedentary- or endurance-trained individuals [15]. This suggests that TMAO is unlikely acutely affected by diet, particularly when TMAO precursors are not consumed. Additionally, TMAO is cleared by the kidney and metabolized in the liver [40]. Although we did not examine kidney or liver function with regard to TMAO, per se, our clinical labs indicate that people had relatively normal kidney and liver function. Further, FMO3 action in the liver mediates the oxidation of TMA to TMAO and is influenced at least in part by liver insulin sensitivity [41]. While we did not measure FMO3 to discern TMA metabolism in the gut and TMAO oxidation in the liver prior to systemic circulation, neither TMA nor TMAO were altered in this study, despite reductions in fasting glucose and insulin. Given the liver is the primary organ regulating fasting glucose homeostasis [42], our work suggests TMAO metabolism is unaltered independent of lower hepatic insulin resistance.

In conclusion, overall, neither LCD nor LCD+INT for 2 weeks was effective at reducing plasma TMAO in women with obesity. However, in women with higher circulating baseline TMAO levels, both treatments lowered plasma TMAO. This is consistent with our observation that LCD, with or without INT treatment, is effective at lowering choline, a key precursor to TMAO. The clinical relevance of lower TMAO metabolism in women with obesity is unclear; however, lower TMA and carnitine concentrations were related to improved central hemodynamics, which may promote CVD risk reduction. Therefore, additional studies are necessary to understand how lifestyle interventions and/or medications that influence the gut may reduce TMAO among individuals with obesity to combat CVD.

## Figures and Tables

**Table 1:** Effect of a low-calorie diet vs. a low-calorie diet and interval exercise on anthropometrics, fitness, and glycemia.

	LCD		LCD+INT		ANOVA *p* Value	
	Pre	Post	Pre	Post	Test	G × T
*n* (female)	12	-	11	-		
Non-Hispanic white	11	-	6	-		
Non-Hispanic black	0	-	3	-		
Hispanic	1	-	1	-		
Asian Pacific Islander	0	-	1	-		
Age, yr	48.4 ± 2.6	-	47.6 ± 4.3	-		
Weight, kg	102.1 ± 5.0	99.7 ± 4.9	104.9 ± 6.9	103.2 ± 6.8	<0.01	0.19
BMI, kg/m^2^	37.8 ± 1.6	37.0 ± 1.6	38.0 ± 2.3	37.4 ± 2.3	<0.01	0.13
Body fat, %	51.5 ± 1.4	50.2 ± 1.5	49.1 ± 2.5	48.2 ± 2.7	<0.01	0.89
FFM, kg	49.4 ± 1.5	49.0 ± 1.3	52.5 ± 2.8	52.7 ± 2.9	0.78	0.45
VO_2_peak, L/min	1.83 ± 0.1	1.65 ± 0.1	1.9 ± 0.1	2.0 ± 0.1	0.61	0.03
VO_2_peak, ml/kg/min	18.1 ± 1.0	17.5 ± 1.1	19.0 ± 1.6	20.3 ± 1.8	0.50	0.05
HDL Cholesterol, mg/dL	50.8 ± 2.2	43.7 ± 2.0	42.9 ±1.7	49.1 ± 4.6	0.06	<0.01
LDL Cholesterol, mg/dL	136.3 ± 13.0	115.0 ± 11.5	114.4 ± 8.2	100.2 ± 5.7	<0.01	0.62
Glucose						
Fasting, mg/dL	97.1 ± 1.5	94.2 ± 2.5	97.0 ± 2.5	92.9 ± 2.2	0.05	0.71
120 min, mg/dL	113.1 ± 6.3	115.3 ± 9.0	112.8 ± 7.0	126.0 ± 8.3	0.66	0.93
tAUC, mg/dlx180 min	20,482.6 ± 965.8	20,550.8 ± 1242.4	22,612.2 ± 1039.3	22,416.7 ± 1245.0	0.91	0.82
Insulin						
Fasting, μU/mL	15.3 ± 2.1	11.8 ± 2.3	22.3 ± 6.2	18.2 ± 4.1	0.03	0.77
120 min, μU/mL	83.2 ± 14.9	74.2 ± 16.0	125.3 ± 20.5	80.2 ± 19.1	0.45	0.68
tAUC, μU/mLx180 min	12,681.0 ± 1794.0	12,737.4 ± 1864.7	19,252.2 ± 2805.5	12,905.5 ± 1043.3	0.01	0.31

Note: Data are mean ± SEM. LCD = low-calorie diet. LCD+INT = low-calorie diet and interval exercise.

**Table 2:** Effect of a low-calorie diet vs. a low-calorie diet and interval exercise on caloric intake, macronutrients, and fiber from 3-day diet logs.

	LCD		LCD+INT		ANOVA *p* Value	
	Pre	Post	Pre	Post	Test	G × T
Total Kcal, kcal	2013.9 ± 191.9	1608.9 ± 88.2	2047.7 ± 191.1	1346.5 ± 29.1	<0.01	0.67
Carbohydrates, g	235.5 ± 25.5	175.2 ± 6.6	238.7 ± 28.8	217.6 ± 13.1	0.05	0.33
Fiber, g	17.3 ± 1.9	10.5 ± 0.7	17.2 ± 1.4	10.1 ± 1.1	<0.01	0.92
Fat, g	86.7 ± 7.7	44.5 ± 2.0	84.1 ± 8.7	49.6 ± 4.3	<0.01	0.51
Protein, g	83.3 ± 8.1	61.8 ± 3.0	78.2 ± 8.9	71.9 ± 3.7	0.03	0.20

Note: Data are mean ± SEM. LCD = low-calorie diet. LCD+INT = low-calorie diet and interval exercise. Pre-test diet was the average of 3 d food logs. Post-test diet was the average of 13 d food logs.

**Table 3:** Effect of a low-calorie diet vs. a low-calorie diet and interval exercise on fasting hemodynamics.

	LCD		LCD+INT		ANOVA *p* Value	
	Pre	Post	Pre	Post	Test	G × T
bSBP						
Fasting, mmHg	131.8 ± 5.6	129.4 ± 4.6	139.5 ± 6.6	137.5 ± 7.8	0.47	0.93
120 min, mmHg	133.3 ± 7.0	130.7 ± 3.6	133.5 ± 4.8	136.1 ± 7.3	0.96	0.36
tAUC, mmHgx180 min	24,577.5 ± 1040.1	23,410.0 ± 712.7	24,256.4 ± 883.9	24,409.1 ± 1271.9	0.97	0.73
bDBP						
Fasting, mmHg	82.8 ± 3.6	77.2 ± 3.5	80.2 ± 3.4	82.0 ± 5.0	0.39	0.11
120 min, mmHg	81.1 ± 3.7	75.8 ± 3.5	77.4 ± 3.7	78.0 ± 3.8	0.37	0.16
tAUC, mmHgx180 min	14,825.0 ± 615.1	13,805.0 ± 562.7	14,075.4 ± 560.2	14,269.1 ± 767.9	0.30	0.07
cSBP						
Fasting, mmHg	122.7 ± 4.9	117.2 ± 2.8	128.0 ± 6.1	126.9 ± 7.5	0.48	0.73
120 min, mmHg	121.2 ± 6.5	118.5 ± 3.4	119.6 ± 5.1	121.1 ± 6.9	0.93	0.44
tAUC, mmHgx180 min	21,309.0 ± 425.3	20,541 ± 312.2	21,867.3 ± 925.3	21,916.4 ± 1210.9	0.41	0.28
cDBP						
Fasting, mmHg	84.0 ± 3.6	78.3 ± 3.5	82.1 ± 3.5	83.2 ± 5.0	0.30	0.15
120 min, mmHg	82.4 ± 4.2	78.5 ± 3.8	76.8 ± 3.4	79.3 ± 4.1	0.31	0.18
tAUC, mmHgx180 min	14,596.4 ± 492.4	14,030.0 ± 564.9	14,350.9 ± 572.6	14,479.1 ± 789.3	0.43	0.26
PPA						
Fasting, mmHg	1.27 ± 0.0	1.24 ± 0.0	1.26 ± 0.0	1.28 ± 0.0	0.90	0.33
120 min, mmHg	1.36 ± 0.0	1.32 ± 0.0	1.38 ± 0.0	1.41 ± 0.1	0.92	0.23
tAUC, mmHgx180 min	238.7 ± 3.67	237.0 ± 5.6	243.3 ± 7.6	243.2 ± 4.5	0.98	0.97
AIx75						
Fasting, %	29.9 ± 3.4	28.0 ± 3.3	29.5 ± 5.7	24.7 ± 6.1	0.28	0.63
120 min, %	24.5 ± 4.7	20.8 ± 3.5	16.4 ± 5.3	17.1 ± 5.5	0.68	0.50
tAUC, %x180 min	4917.5 ± 524.8	3830.0 ±576.6	3927.0 ± 856.6	3645.0 ± 911.9	0.08	0.30
AP						
Fasting, mmHg	13.5 ± 1.0	15.1 ± 1.8	15.4 ± 2.8	13.6 ± 2.9	0.96	0.30
120 min, mmHg	10.9 ± 2.0	11.3 ± 1.9	8.5 ± 2.4	8.8 ± 2.4	0.82	0.99
tAUC, mmHgx180 min	2337.5 ± 225.9	2077.5 ± 317.5	1956.0 ± 426.9	1713.0 ± 414.7	0.19	0.96
Pf						
Fasting, mmHg	26.5 ± 1.1	26.1 ± 1.7	31.4 ± 2.3	25.6 ± 1.9	0.04	0.27
120 min, mmHg	27.3 ± 1.7	27.4 ± 1.1	26.5 ± 1.2	30.0 ± 2.9	0.36	0.36
tAUC, mmHgx180 min	4848.4 ± 131.1	4628.0 ± 208.6	5335.3 ± 268.4	5382.3 ± 241.9	0.55	0.30
Pb						
Fasting, mmHg	17.1 ± 1.0	18.2 ± 1.2	20.7 ± 1.6	19.4 ± 2.4	0.93	0.56
120 min, mmHg	16.7 ± 1.7	18.3 ± 1.2	19.1 ± 1.0	18.2 ± 2.2	0.81	0.36
tAUC, mmHgx180 min	3027.4 ± 141.8	3015.7 ± 214.9	3443.0 ± 219.5	3523.5 ± 305.6	0.29	0.73
RM						
Fasting, %	65.3 ± 4.3	70.3 ± 4.4	65.8 ± 3.0	67.9 ± 5.3	0.45	0.68
120 min, %	60.7 ± 3.8	63.4 ± 3.1	69.5 ± 4.5	60.2 ± 4.9	0.33	0.13
tAUC, %x180 min	11,146.7 ± 512.1	11,566.7 ± 458.0	11,139.0 ± 466.4	10,941.4 ± 877.7	0.88	0.51
HR						
Fasting, bpm	64.3 ± 2.0	60.3 ± 1.3	68.8 ± 3.0	66.9 ± 3.3	0.08	0.52
120 min, bpm	68.9 ± 1.6	62.4 ± 2.0	71.2 ± 2.4	72.3 ± 3.2	0.21	0.09
tAUC, bpmx180 min	12,167.5 ± 237.8	11,350.0 ± 261.0	12,248.2 ± 584.5	12,585.0 ± 499.0	0.55	0.05

Note: Data are mean ± SEM. LCD = low-calorie diet. LCD+INT = low-calorie diet and interval exercise. bSBP = brachial systolic blood pressure. bDBP = brachial diastolic blood pressure. cSBP = central systolic blood pressure. cDBP = central diastolic blood pressure. PPA = pulse pressure amplification. AIx75 = augmentation index corrected to 75 bpm (heart rate). AP = augmentation pressure. Pf = forward pressure. Pb = backward pressure. RM = reflection magnitude. HR = heart rate.

**Table 4:** Effect of a low-calorie diet vs. a low-calorie diet and interval exercise on fasting plasma trimethylamine N-oxide precursors.

	LCD		LCD+INT		ANOVA *p* Value	
	Pre	Post	Pre	Post	Test	G × T
TMA (μM)	16.9 ± 1.0	16.5 ± 1.3	15.6 ± 0.8	15.6 ± 1.0	0.62	0.62
Carnitine (μM)	35.6 ± 2.0	35.2 ± 2.3	33.4 ± 1.6	33.6 ± 1.9	0.89	0.67
Choline (μM)	8.0 ± 0.5	6.6 ± 0.6	9.7 ± 0.6	7.9 ± 0.3	<0.01	0.60
Betaine (μM)	22.9 ± 1.8	22.6 ± 1.9	24.0 ± 1.6	23.3 ± 1.8	0.54	0.80

Note: Data are mean ± SEM. LCD = low-calorie diet. LCD+INT = low-calorie diet and interval exercise. TMA = trimethylamine.

## Data Availability

Data is available from the corresponding author upon reasonable request.

## Clinical Trials Registration

NCT03355469.
